# Even subtle cultural differences affect face tuning

**DOI:** 10.1371/journal.pone.0198299

**Published:** 2018-06-11

**Authors:** Marina A. Pavlova, Julie Heiz, Alexander N. Sokolov, Andreas J. Fallgatter, Koviljka Barisnikov

**Affiliations:** 1 Department of Psychiatry and Psychotherapy, Medical School, Eberhard Karls University of Tübingen, Tübingen, Germany; 2 Child Clinical Neuropsychology Unit, Department of Psychology, University of Geneva, Geneva, Switzerland; 3 Department of Women’s Health, Women’s Health Research Institute, University Hospital, Eberhard Karls University of Tübingen, Tübingen, Germany; Tilburg University, NETHERLANDS

## Abstract

Culture shapes social cognition in many ways. Yet cultural impact on face tuning remains largely unclear. Here typically developing females and males from the French-speaking part of Switzerland were presented with a set of Arcimboldo-like Face-n-Food images composed of food ingredients and in different degree resembling a face. The outcome had been compared with previous findings obtained in young adults of the South-West Germany. In that study, males exhibit higher thresholds for face tuning on the Face-n-Food task than females. In Swiss participants, no gender differences exist in face tuning. Strikingly, males from the French-speaking part of Switzerland possess higher sensitivity to faces than their German peers, whereas no difference in face tuning occurs between females. The outcome indicates that even relatively subtle cultural differences as well as culture by gender interaction can modulate social cognition. Clarification of the nature of cultural impact on face tuning as well as social cognition at large is of substantial value for understanding a wide range of neuropsychiatric and neurodevelopmental conditions.

## Introduction

Culture sculpts human cognition and, in particular, social cognition in many ways [1]. Previous work has established cultural differences in the general style of how Easterners and Westerners perceive their visual world: Easterners attend to the visual world more broadly [2,3]. It is widely assumed that these differences reflect the mere use of holistic processing of visual input by Easterners and analytical processing by Westerners. During face processing, Easterners tend to fixate toward the center of the face (a nose) even if they need information provided at periphery, i.e. by eyes and a mouth [4–9]. This tendency in eye fixation still exists when faces are inverted in the image plane [10]. The differences in fixation patterns, however, may reflect cultural differences in the socially acceptable duration of eye contact rather than cultural influences on the visual mechanisms underlying face encoding [11]. More recent data show that cultural differences occur already at early stages of face processing: despite comparable low-level contrast sensitivity functions, Easterners (Chinese) are reported to be more tuned toward lower spatial frequencies than Westerners (Canadians) [12].

It is well-known that perception and rating of facial emotional intensity varies across cultures, and it is largely modulated by socio-cultural norms [13,14]. Furthermore, facial expressions of basic emotions are not culturally universal [15]. Culture touches not only behavioral but also neural response for faces. Overall, Westerners engage greater object-processing activity while East Asians engage more context-processing activity in the ventral visual areas of the brain [16]. In Westerners, the left fusiform face area (FFA) exhibits more selectivity for faces, whereas Easterners show right lateralization of the FFA activity [17]. Cultural Neuroscience points to stronger amygdala activation in response to out-group faces [18–23]. Yet this effect is modulated by expressed emotion, for instance, by fear: native Japanese in Japan and Caucasians in the United States show greater amygdala activation to fear expressed by members of their own ethnical/cultural group [24]. This suggests that the amygdala tends to respond to facial expressions typical for one’s own cultural group. For fearful faces, the right inferior frontal gyrus, premotor cortex and left insula are engaged in Japanese observers, whereas Caucasians activate the posterior cingular cortex, supplementary motor cortex, and the left amygdala [25]. It appears that Caucasians respond to fearful faces in a more direct and emotional way. In both Caucasian American and Japanese students, during the averted vs. direct gaze, elevated amygdala activation is observed when fearful face expressions are portrayed by in-group actors; during direct vs. averted gaze, stronger amygdala activation is found when fear is expressed by out-group actors [26]. Electrophysiological data investigating own-race bias effects in face recognition indicates a finer-grained neural tuning for same-race faces [27]. This effect occurs already at early stages of face processing: inverted same-race faces elicit larger N170 component along with better face recognition in both Western Caucasian and East Asian observers [28]. Exploration of the ‘alien-effect’ (hitches in understanding emotional face expressions of individuals of different cultural backgrounds, leading to lower recognition accuracy and stronger amygdala activation) in Asian and European students by using Caucasian facial expressions shows that culture and duration of stay in a foreign culture are influential factors at both behavioral and brain levels [22–23], whereas gender of perceivers produces only a subtle effect [23]. The outcome of these studies also demonstrate cultural influence on recognition of disgust and anger from facial expressions: in the Asian sample, recognition accuracy is lower.

As described above, most studies on cultural effects on social cognition have been conducted in populations with pronounced cultural differences such as Easterners and Westerners. However, more subtle cultural differences may also affect social cognition. For instance, comparison of performance on neurocognitive tasks in 3- to 15- year-old children from Western countries (Finland, Italy, and the United States of America) indicates that Italians exhibit higher scores on affect recognition which is an essential component of social competence [29]. Within-Asian differences in facial anger recognition are also reported. When presented with Japanese and Caucasian facial expressions of anger, Chinese individuals perform significantly better than Japanese participants; Japanese observers exhibit a stronger tendency not to label negative emotional face expressions as negative, but rather as neutral [30]. (Yet, cultural differences in emotion recognition can generally reflect differences in emotion expression.)

**Fig 1:**
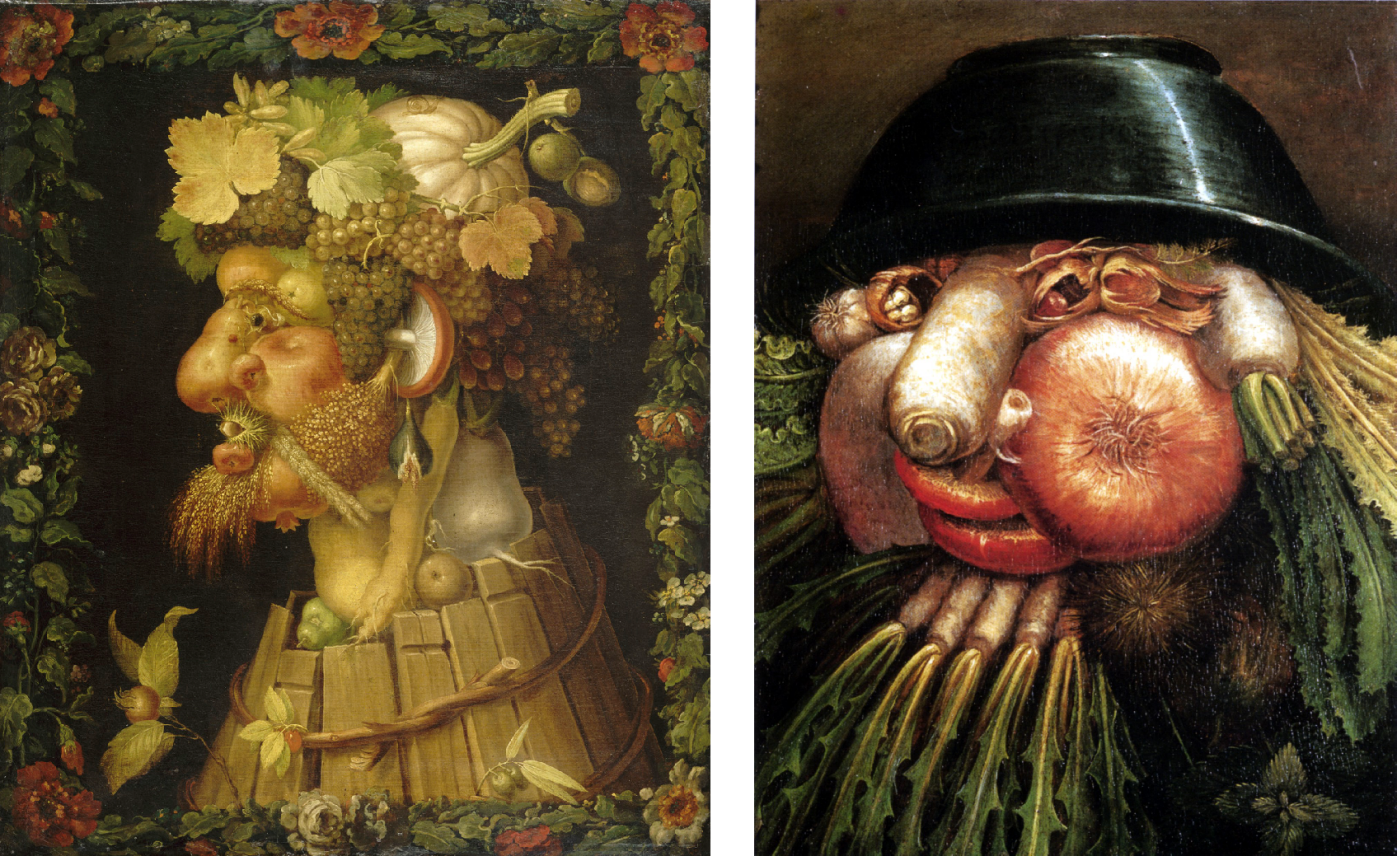
Examples of the Giuseppe Arcimboldo style. ‘Autumn’ (left) and ‘The Greengrocer’ (right) by Guiseppe Arcimboldo (1526–1593), an Italian painter best known for creating fascinating, often grotesque and allegoric, imaginative portraits composed of fruits, vegetables, plants, tree roots, flowers, and even books and human/animal bodies (https://commons.wikimedia.org/wiki/Giuseppe_Arcimboldo; public domain).

**Fig 2:**
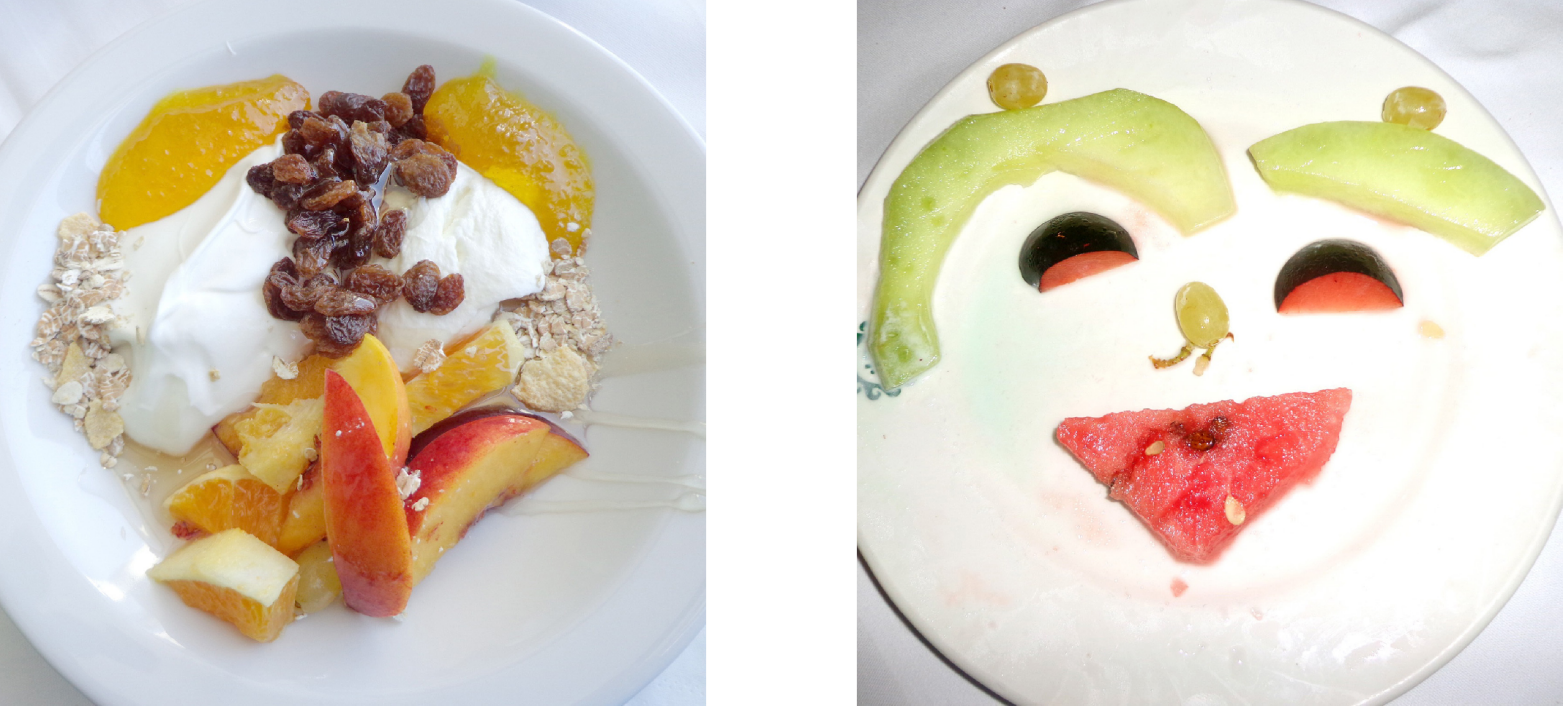
Examples of images used. The least resembling face (left panel) and most resembling face (right panel) images from the Face-n-Food task (from Pavlova, M.A., Scheffler, K., Sokolov, A.N. 2015. Face-n-Food: Gender Differences in Tuning to Faces. PLoS ONE 10(7): e0130363. doi:10.1371/journal.pone.0130363; the Creative Commons Attribution (CC BY) license).

The goal of the present work is uncovering of potential culture effects (within Western population) on face tuning in a recently established Face-n-Food task. As described earlier [31–34], this task consists of a set of food-plate images composed of food ingredients (fruits, vegetables, sausages, etc.) in a manner slightly bordering on the style of Giuseppe Arcimboldo (1526–1593), an Italian painter best known for producing fascinating imaginative portraits composed entirely of fruits, vegetables, plants and flowers (Fig 1). One can perceive a Face-n-Food image either as a number of elements (fruits, vegetables, etc.) or as a face (Fig 2). The foremost benefit of these images is that single components do not explicitly trigger face-specific processing, whereas in face images commonly used for investigating face perception (such as photographs), the mere occurrence of typical features or cues (such as a nose or mouth) already implicates face presence [33–34]. This task also profits from using unfamiliar images that is of special value in clinical settings and cross-cultural studies [35].

In this study, the Face-n-Food task had been administered to young female and male adults, students of the University of Geneva, the French-speaking part of Switzerland. We asked whether in this population gender differences occur on the Face-n-Food task. Our previous work showed gender differences in tuning to faces in students of the University of Tübingen, the South-West Germany [31]. Our further intention, therefore, was to compare face sensitivity in participants of Switzerland and Germany with the purpose to clarify whether even subtle cultural differences can potentially affect face tuning.

## Methods

### Participants

Sixty-two young adults (32 females, 30 males), students of the University of Geneva, Switzerland were enrolled in the study. Participants were aged 24.5±0.94 years (median±95% confidence interval): females, 24±1.5 years (age range, 22 to 45 years); and males 26±1.16 years (age range, 22 to 36 years). There was no age difference between them (Mann-Whitney test, *U* = 1.93, n.s.). They were run individually. All of them had normal or corrected-to-normal vision. None had a history of neurological or psychiatric disorders, nor regular drug intake (medication). They were naïve as to the purpose of the study, and none had previous experience with such images and tasks. Participants were recruited from Social Sciences Schools. The study was conducted in line with the Declaration of Helsinki and was approved by the Ethics Committee of the Department of Psychology and Educational Sciences at the University of Geneva, Switzerland. Informed written consent was obtained from all participants. Participation was voluntary, and the data were processed anonymously.

### The Face-n-Food task: Design and procedure

The Face-n-Food task was administered to participants. This task is described in detail elsewhere [31–34]. In brief, a set of ten images had been created that were composed of food ingredients (fruits, vegetables, sausages, etc.), and to different degree resembled faces. The images slightly border on the Giuseppe Arcimboldo style (Figs 1 and 2). Participants were presented with the set of images, one by one, in the predetermined order from the least to most resembling a face (images 1 to 10). This order was determined in the previous study with typically developing adult volunteers [34]. The order had been used since once seen as a face, Face-n-Food images are often processed with a face-dominating bias. On each trial, participants had to perform a spontaneous recognition task: they were asked to briefly describe what they saw. Their reports were recorded, and then analyzed by independent experts. For further data processing, the responses were coded as either non-face (0) or face (1) report. To avoid time pressure that can potentially cause stress and negative emotional and physiological reactions blocking cognitive processes, there was no time limit on the task. With each participant, the testing procedure lasted for about 20–25 min. The data analysis was conducted by using statistical methods with the software package JMP (V13.1 SAS Institute Inc. 2017; Cary, NC).

## Results

Participants were presented with the set of Face-n-Food images, one by one, in the predetermined order from the least to most resembling a face (images 1 to 10). They described a food-plate image either in terms of food composition (non-face response, 0) or as a face or smiley (face response, 1). When an image had been seen as a face, perceivers often provide interpretations in emotional terms (e.g., *c’est qqun qui sourit*, *qui a l’air heureux—das ist jemand der lacht*, *der sieht glücklich aus—it is someone who smiles*, *who looks happy*). As in the earlier studies with typically developing young adults and patients [31–34], responses other than face or food were given extremely rarely, and in accord with our binomial classification were coded as non-face reports (0).

**Fig 3:**
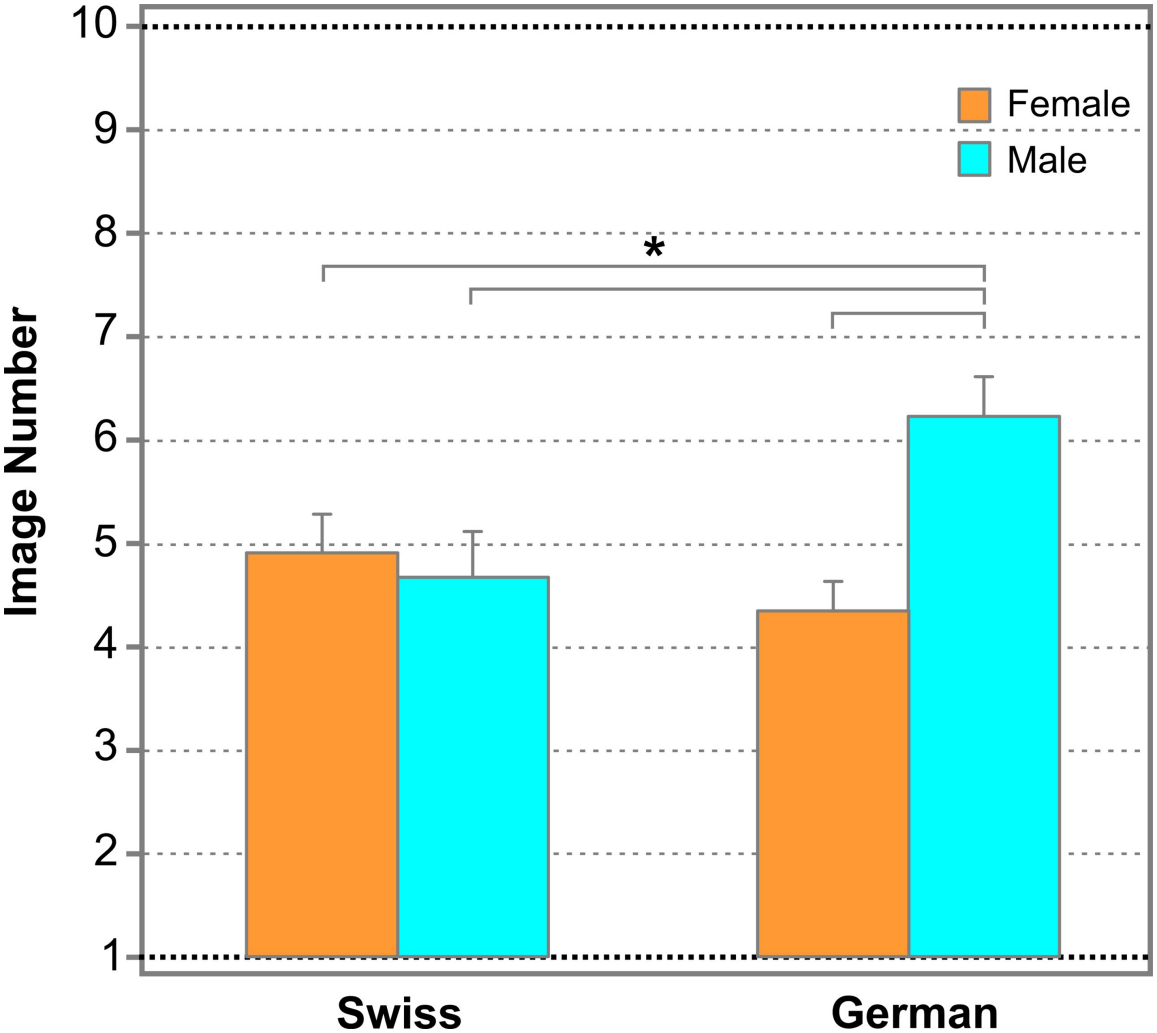
Tuning to faces. The average image number, on which resembling a face on the Face-n-Food task (face response) was initially reported, separately for female and male Swiss and German participants. The data for German participants had been reported earlier (Pavlova, M.A., Scheffler, K., Sokolov, A.N. 2015. Face-n-Food: Gender Differences in Tuning to Faces. PLoS ONE 10(7): e0130363. doi:10.1371/journal.pone.0130363). Significant difference in thresholds for face tuning between different groups of participants is indicated by asterisk and horizontal bars. Vertical bars represent SEM.

Fig 3 shows the thresholds for face tuning (i.e., average image number, on which face response was initially reported on the Face-n-Food task) separately for female and male participants of the French speaking part of Switzerland and South-West Germany. By contrast with German peers [31], there were no gender differences in face sensitivity in Swiss participants: females reported seeing a face on 4.94±2.23 (mean±SD) and males on 4.7±2.25 image (t(60) = 0.46, p = 0.678, n.s.).

As seen from Fig 3, male participants from the South-West Germany experience more troubles in spontaneous face recognition than their peers from the French-speaking part of Switzerland and German females. In general, they need a more salient image to seeing a face than their peers. Face tuning of all other groups is comparable with each other. As reported earlier [31], German males exhibit higher thresholds for faces on the Face-n-Food task than females, that is, they reported seeing a face later. Individual data (image number, on which face response was reported for the first time) of all perceivers were submitted to a two-way analysis of variance, ANOVA, with factors Gender (male/female) and Culture (Swiss/German). The outcome did not reveal any main effect of Culture (F(1;123) = 1.8, p = 0.18, n.s.), whereas main effect of Gender was significant (F(1;123) = 5.24, p < 0.024, with an effect size η2 = 0.041). The Gender-by-Culture interaction was also significant (F(1;123) = 8.63, p < 0.004, with an effect size η2 = 0.065). Post-hoc analysis revealed that, as expected, the difference was significant between German and Swiss males (t(58) = 2.57, p < 0.006; with an effect size Cohen’s d = 0.731), and between German males and Swiss females (t(60) = 2.17, p < 0.017; with an effect size Cohen’s d = 0.621). No difference was found between German and Swiss females (t(64) = 0.72, p = 0.236; n.s.).

Once seen as a face, Arcimboldo-like paintings are often perceived with a face-dominating bias [36]. However, from time to time both typically developing individuals and patients give non-face responses on subsequent Face-n-Food images [31,33,34]. As some perceivers of the present study as well as participants of our earlier study [31] did not report seeing a face on all subsequent images after the first face response, we performed additional analyses on percentage of images recognized as a face. In agreement with the previous analysis performed on initial face responses (see above), no gender differences in face tuning was found in Swiss participants: the percentage of face responses was 55.63±19.99 (mean±SD) in females and 57.67±22.08 in males (t(60) = 0.54, p = 0.705, n.s.). A two-way ANOVA with factors Gender (male/female) and Culture (Swiss/German) performed on individual percentage of face-responses shows that the main effect of Culture was not significant (F(1;123) = 0.856, p = 0.36, n.s.), whereas the main effect of Gender approached significance (F(1;123) = 3.396, p < 0.068). The Culture-by-Gender interaction was significant (F(1;123) = 5.9, p < 0.017, with an effect size η2 = 0.045). Post-hoc analysis revealed that no difference occurred between German and Swiss females (t(64) = 0.74, p = 0.232; n.s.), though the difference in the percentage of face responses between German and Swiss males was significant (t(58) = 2.04, p < 0.023; with an effect size Cohen’s d = 0.527). This difference cannot be explained simply by stronger experimenter expectancy (when participants wish to please the experimenter) or by entire bias towards seeing faces in Swiss males, because 13 out of 30 (43%) Swiss males gave at least one non-face response on subsequent images after the first face response. By contrast, only 3 out of 30 (10%) German males reported seeing food elements or something different as a face on subsequent images after the first face response [31]. The difference between German and Swiss males in the number of non-face responses after the first face response was significant (Mann-Whitney, *U* = 2.18, p < 0.029, with an effect size η2 = 0.731).

**Fig 4:**
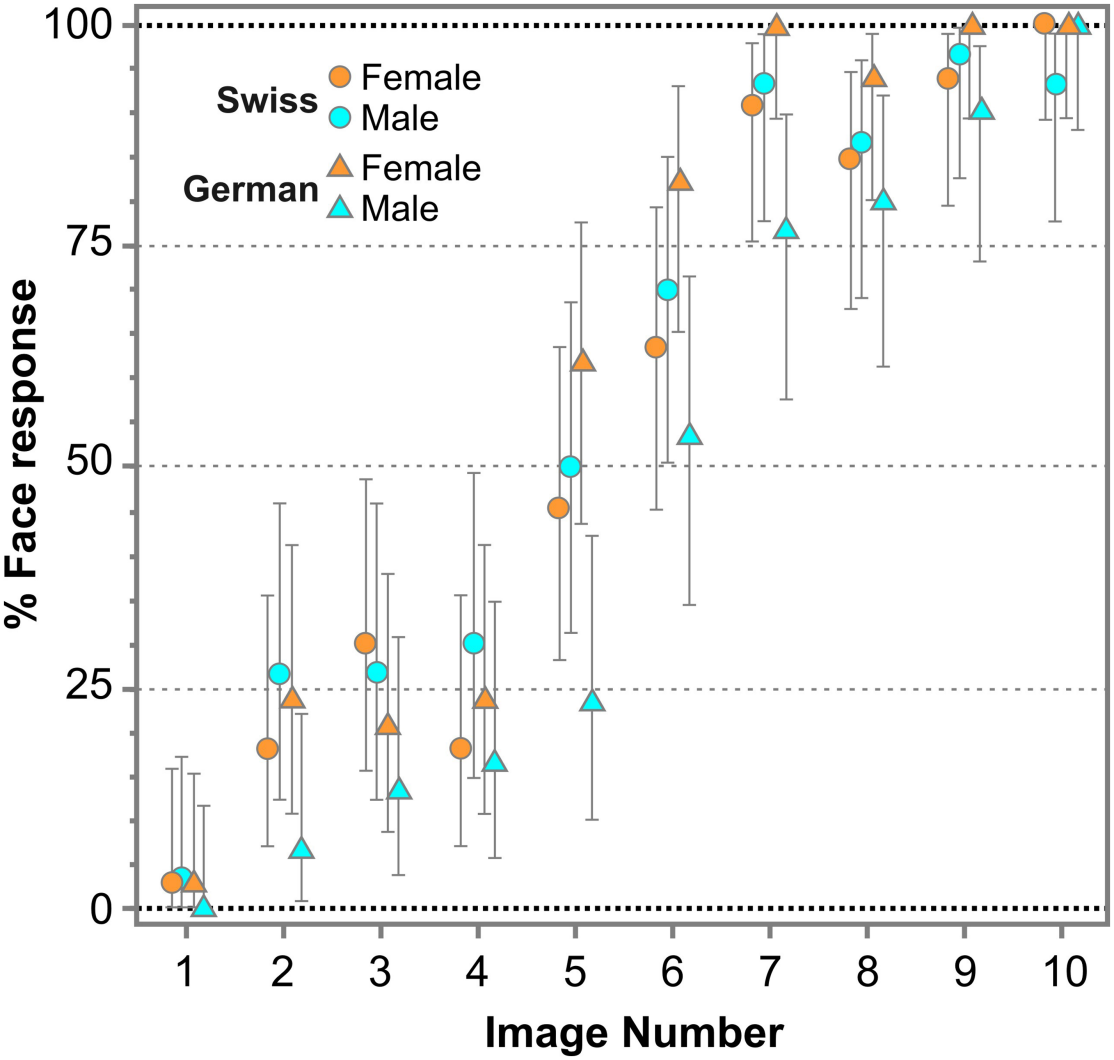
Percentage of face responses for each Face-n-Food image. The image number reflects its face resemblance (1 –the least recognizable, 10 –the most recognizable as a face). Vertical bars represent 95% confidence interval, CI. The data for German participants had been reported earlier (Pavlova, M.A., Scheffler, K., Sokolov, A.N. 2015. Face-n-Food: Gender Differences in Tuning to Faces. PLoS ONE 10(7): e0130363. doi:10.1371/journal.pone.0130363).

Fig 4 represents the percentage of face responses for each Face-n-Food image separately for female and male participants of the present study in Swiss participants and previous study conducted in the South-West Germany [31]. As indicated by multiple stepwise nominal logistic regression analysis performed on the number of face responses given for each Face-n-Food image for German and Swiss females and males, the interaction between Culture and Image is significant (χ^2^(1) = 4.011, p < 0.045, with an effect size η2 = 0.0318): this means that the fitted face recognition curves are not parallel. This indicates that the face recognition dynamics is different, and spontaneous face recognition is not uniformly shifted down in the group with lower face tuning. Most pronounced discrepancy in face sensitivity occurs on intermediate images 5–6: German males provided (i) only 25% face responses on image 5, whereas the other groups of participants gave about 50% or even more face responses; and (ii) slightly more than 50% on image 6, whereas the others gave about 65–80% face responses.

## Discussion

The present work was aimed at investigation of potential culture effects (within Western population) on face tuning in a recently established Face-n-Food task. The task had been administered to young female and male adults, students of the University of Geneva, the French-speaking part of Switzerland, and further compared with earlier findings obtained in students of the University of Tübingen, the South-West Germany [31]. In that study, males exhibited higher thresholds for face tuning on the Face-n-Food task than females. Here we asked (i) whether gender differences occur on this task in Swiss population; and (ii) whether even subtle cultural differences (between young females and males of the French-speaking part of Switzerland and South-West Germany) affect face tuning on this task. We were also interested in the culture-by-gender interaction.

The outcome shows that in Swiss participants, no gender difference occurs in face tuning. Strikingly, males from the French-speaking part of Switzerland possess higher sensitivity to faces than their German peers, whereas no difference in face tuning exists between females of both countries. Moreover, all other groups of participants (Swiss females and males, and German females) performed on the Face-n-Food task at approximately the same level. The findings indicate that even relatively subtle cultural differences as well as culture by gender interaction affect such essential component of social cognition as tuning to faces.

The question arises: What are the origins of lower face sensitivity on the Face-n-Food task? One possible explanation is that poorer performance may be caused by general (non-face-specific) difficulties in visual feature integration. Indeed, one can perceive a Face-n-Food image either as a composition of local elements (fruits, vegetables, etc.) or as a Gestalt. Once seen as a face, the Face-n-Food images are processed with a strong face-dominating bias [33,34] and, therefore, top-down influences may affect bottom-up processing of these images. In line with this, recent findings indicate that original Arcimboldo ‘hidden-face’ portraits are judged as being more ambiguous by perceivers with local than with holistic perceptual style [36,37]. This suggests that perceivers with holistic style see faces in these portraits more easily. Moreover, females (but not males) with a local perceptual style judge Arcimboldo artwork as more aesthetically pleasant than do their peers with holistic style [36]. It is known that ambiguous artwork (though it is harder to process) can be associated with greater pleasure and curiosity [38]. On the other hand, by females with holistic perceptual style (who perceive faces in Arcimboldo portraits effortlessly) these paintings may be experienced as unpleasant since the depicted faces are sometimes bizarre and ugly. In accord with this, greater activation of the occipito-temporal brain network dedicated to face encoding (including the fusiform gyri, parahippocampal gyri, and the inferior temporal gyri) is observed when perceivers are displeased by Arcimboldo artwork [37]. Other experimental data also suggests that intact visual feature integration (leading to holistic perception) is required for recognition of Arcimboldo-like faces. Face sensitivity to Arcimboldo paintings emerges early in typical development: Japanese infants aged 7–8 months prefer the Arcimboldo portraits over the same paintings presented upside-down (stimulus inversion impairs proper feature integration, and makes these portraits difficult to perceive as a face) [39]. Eye-tracking in Westerners shows that with Arcimboldo portraits (similarly with other faces), perceivers tend to fixate more the upper (‘eyes’) part of upright paintings, whereas the lower part (‘mouth’) corresponding to the position of eyes in upright stimuli is fixated with inverted orientation [40]. Pronounced difficulties in seeing faces on the Face-n-Food task are reported in individuals with Williams-Beuren syndrome (examined in the French speaking part of Switzerland and France) [33] and individuals with autistic spectrum disorders (tested in the North Italy) [34]: this scarcity may be accounted for, at least, partly, by deficits in visual feature integration. Yet, patient SL with visual simultanagnosia (that is characterized by focusing on visual analysis of local elements along with difficulties in perceiving wholes) perceives Arcimboldo paintings as faces without any troubles [41].

The alternative explanation for lower face sensitivity may be of socio-cultural origin. Due to some reasons (such as differences in upbringing and socially desirable gender-specific behavioral styles), males of the French-speaking part of Switzerland may possess more sensible personality profile that is manifested in more pronounced attentiveness to social interaction and communication including faces. As seen in Fig 4, the core difference in the face recognition dynamics is that German males provide fewer face responses not only on the first images that are difficult to integrate and recognize, but also on the last, easily recognizable, almost ‘pop-out’ images requiring much less perceptual-attentional resources. Most troubles they experience with the intermediate images 5 and 6. This suggests that interplay of factors (e.g., lower drive to social stimuli such as faces in addition to possible difficulties in visual feature integration) may be responsible for poorer face tuning in this sample of participants. Since experimental clarification of this issue is beyond the scope of the present study, future efforts should be aimed at investigation of the origins of cultural differences in face sensitivity.

Brain imaging can substantially contribute to clearing up the nature of variability in face tuning: it can help to clarify whether differences in topography and activation of the face-sensitive brain networks exist between perceivers with high and low face sensitivity. To the date, brain imaging and neuropsychological work dedicated to visual processing of Arcimboldo portraits is rather scarce and controversial. Arcimboldo paintings compared to Renaissance portraits and photographs of natural faces lead to greater functional magnetic resonance imaging (fMRI) activation in the occipito-temporal network (including the FFA) underpinning face processing, but also in the right inferior frontal gyrus and bilateral superior and inferior parietal lobule [37] that might indicate involvement of attention. Yet patient GG with prosopagnosia (inability to recognize faces properly) following unilateral right brain damage is reported to be capable of perceiving Arcimboldo portraits [42]. Compared to the same inverted displays that are much less recognizable as faces, upright Arcimboldo portraits activate the right middle fusiform gyrus (including FFA) and posterior superior temporal sulcus (pSTS) [43]. In accord with this, electroencephalography (EEG) indicates that face-sensitive N170 component of the event-related potentials (ERP) in the right occipito-temporal region does not differ between upright Arcimboldo portraits and natural faces, whereas in the left hemisphere, it does [44]. This suggests that the right hemisphere is involved in holistic perception of Arcimboldo faces, whereas the left hemisphere is rather responsible for processing of local features. For upside-down orientation, N170 amplitude in both hemispheres is the same in response to Arcimboldo paintings and familiar objects (such as car and house depictions), but it is reduced compared to natural faces. Disruption of holistic processing with inversion may lead to perception of Arcimboldo portraits as a mere composition of elements (fruits, vegetables, etc.). By contrast with EEG findings, however, near-infrared spectroscopy (NIRS) in 7–8 month-old infants indicates that the left temporal area of the brain is more sensitive to the Arcimboldo portraits than to single elements (vegetables) [39]. As indicated earlier [34], a closer look at specific topographic patterns and temporal dynamics of the neural circuitry underpinning facial processing (with hubs in the FFA and pSTS, pivots of the social brain) can add essential information on processing of the Face-n-Food images. For uncovering neural mechanisms of face processing, one may take an advantage of (ultra-)high field fMRI providing for high sensitivity and spatial resolution along with EEG recording to simultaneously obtain precise spatial and temporal information.

Many neuropsychiatric and neurodevelopmental conditions are characterized by impairments in visual social cognition, in particular, in body language reading and facial assessment of a social counterpart [45–52]. In turn, most diseases characterized by aberrant visual social cognition are gender-specific: females and males are differently affected in terms of clinical picture, prevalence, and severity [53,54]. The essential issue of how culture shapes face processing in mental disorders has been often overlooked in rigorous experimental research [55–57]. The culture-by-gender interaction in neuropsychiatric disorders is also largely under-investigated. Cultural differences are more evident in patients than in typically developing individuals, in particular, in processing of non-familiar faces of out-groups [57]. For instance, among American, German and Indian individuals with schizophrenia, Indian patients performed substantially worse on an emotion discrimination task with Caucasian faces [58]. In line with this, both Caucasian- and African-American individuals with schizophrenia are more likely to recognize same-race than other-race faces [59]. The Face-n-Food task that has been shown here to be sensitive even to subtle cultural differences, may serve a valuable tool for uncovering cultural and gender-related alterations in face tuning in neuropsychiatric disorders. This task is not time consuming; and it benefits from using unfamiliar images (not immediately triggering face processing) that is of importance in clinical settings and cross-cultural studies.

## Resume

Most studies on culture effects in social cognition have been conducted in populations with quite pronounced cultural differences such as Easterners and Westerners. The present work was aimed at investigation of potential culture effects on face tuning in a recently established Face-n-Food task within Western population. The Face-n-Food task had been administered to young female and male adults, students of the University of Geneva, the French-speaking part of Switzerland. The findings had been further compared with earlier data obtained in the South-West Germany [31]: in that study, males exhibited higher thresholds for face tuning on the Face-n-Food task than females. The outcome indicates that, by contrast with their German peers, no gender difference exists in face sensitivity in perceivers of the French-speaking part of Switzerland. Strikingly, Swiss males exhibit higher sensitivity to faces than their German peers, whereas no difference exists between females. Moreover, all other groups of participants performed on the Face-n-Food task at approximately the same level. The study indicates that even relatively subtle cultural differences as well as culture-by-gender interaction profoundly modulate such essential component of social cognition as face tuning. The origins of culture influence on face sensitivity should be further investigated by using brain imaging tools. Clarification of the precise nature of cultural impact on face tuning is of substantial value for better understanding a wide range of neuropsychiatric disorders.
